# Implications of GLP-1 Receptor Agonist on Thyroid Function: A Literature Review of Its Effects on Thyroid Volume, Risk of Cancer, Functionality and TSH Levels

**DOI:** 10.3390/biom14060687

**Published:** 2024-06-13

**Authors:** Stefania Capuccio, Sabrina Scilletta, Francesca La Rocca, Nicoletta Miano, Maurizio Di Marco, Giosiana Bosco, Francesco Di Giacomo Barbagallo, Roberto Scicali, Salvatore Piro, Antonino Di Pino

**Affiliations:** Department of Clinical and Experimental Medicine, University of Catania, 95122 Catania, Italy; stefania.cap@hotmail.com (S.C.); sabrinascilletta@gmail.com (S.S.); francescalarocca94@hotmail.it (F.L.R.);

**Keywords:** GLP-1 receptor agonists, thyroid cancer, hypothyroidism, hyperthyroidism, TSH, diabetes

## Abstract

The increasing utilization of Glucagon-like Peptide-1 receptor agonists (GLP-1 RAs) in managing type 2 diabetes mellitus has raised interest regarding their impact on thyroid function. In fact, while these agents are well known for their efficacy in glycemic control and weight management, their association with thyroid disorders requires clarification due to the complex interplay between thyroid hormones and metabolic pathways. Thyroid dysfunction commonly co-occurs with metabolic conditions such as diabetes and obesity, suggesting a profound interconnection between these systems. This review aims to contribute to a deeper understanding of the interaction between GLP-1 RAs and thyroid dysfunction and to clarify the safety of GLP-1 RAs in diabetic patients with thyroid disorders. By synthesizing existing evidence, this review highlights that, despite various studies exploring this topic, current evidence is inconclusive, with conflicting results. It is important to note that these drugs are relatively recent, and longer-term studies with larger sample sizes are likely needed to draw clearer conclusions. Currently, no existing guidelines provide definitive directions on this clinical issue; however, it is advisable to include thyroid function tests in the routine screening of diabetic patients, particularly those treated with GLP-1 Ras, with the goal of optimizing patient care and management.

## 1. Introduction

The remarkable growth in the use of Glucagon-Like Peptide-1 receptor agonists (GLP-1 RAs) in recent years has drawn attention to a class of drugs that have proven to be highly effective in managing type 2 diabetes mellitus (T2DM) and mitigating the effects of its numerous complications. 

These drugs, acting as agonists of Glucagon-like Peptide-1 receptor (GLP-1R), are recognized for promoting insulin secretion, suppressing glucagon release, and delaying gastric emptying [1]. However, while the beneficial effects of GLP-1 RAs on glycemic control, weight loss, and blood pressure reduction are extensively documented, a crucial aspect requiring further investigation is their impact on thyroid function. This interest becomes particularly significant in the context of the intricate interactions involving thyroid hormones (TH) and their interconnection with metabolic pathways, considering the essential role of TH in regulating cellular metabolism. Thyroid diseases frequently emerge in metabolic conditions such as T2DM and obesity, highlighting a complex connection between thyroid dysfunction and metabolic disorders. This association is particularly evident in patients with type 1 and T2DM since epidemiological studies show a significant increase in the prevalence of thyroid dysfunction in these populations, suggesting that the management of metabolic diseases could directly impact thyroid health [2]. 

Studies in animal models have revealed that the use of GLP-1 RAs causes abnormal alterations in thyroid C cells, with a gradual formation of hyperplasia and adenomas [3]. Despite initial reports of a potential increased risk of thyroid cancer in patients treated with GLP-1 RAs, long-term results from clinical studies have alleviated such concerns. The contradiction in these data leaves the discussion open concerning the effect of GLP-1 RAs on the thyroid, emphasizing the need for a systematic investigation. 

Considering the frequent occurrence of thyroid disorders in metabolic diseases such as T2DM and obesity, this scientific review aims to explore in-depth and critically assess the link between the use of GLP-1 RAs and the onset of thyroid disorders. This approach will be implemented through meticulous and detailed analysis of the literature, with the goal of contributing to a more comprehensive understanding of this complex interaction and providing clarity on the safety of GLP-1 RAs in patients with thyroid dysfunction. Additionally, it aims to evaluate the necessity to conduct periodic thyroid function screenings in diabetic patients using GLP-1 agonists.

## 2. Relationship between Type 2 Diabetes Mellitus and Thyroid Function

Thyroid dysfunction and diabetes mellitus are conditions frequently seen in clinical practice and often coexist. Compared with those patients without diabetes, both hyperthyroidism and hypothyroidism are more common in patients with T2DM [4]. 

The prevalence of thyroid dysfunction is significantly higher among patients with T2DM compared with the general population [5]; Perros et al. have reported an overall prevalence of thyroid disease of 13.4% on a total of 1310 adult diabetic patients [6]. A similar prevalence of thyroid dysfunction was found in other studies conducted on Greek (12.3%) [7], Saudi Arabian (16%) [8], Brazilian (14.7%) [9], and Jordanian diabetic populations (12.5%) [10]. Finally, a systematic review reported the association between subclinical hypothyroidism and T2DM with an estimated prevalence of 10.2% [11].

Increased interest has recently focused on the relationship between thyroid function and metabolic diseases. The pathogenetic reasons underlying this interplay are not yet fully understood, but various hypotheses have been formulated. Thyroid hormones influence the regulation of glucose and lipid metabolism, targeting several organs such as the liver, skeletal muscle, pancreas, adipose tissue, and central nervous system [12].

Furthermore, some observational studies suggest an association between T2DM and thyroid cancer, especially in women. Yeo et al. [13] performed a systematic review and meta-analysis to investigate this association, showing that patients with diabetes were at increased risk of cancer by approximately 20%. Insulin resistance, dysglycemia, high body mass index (BMI), and hypertension were shown to significantly increase the incidence of thyroid cancer. Hyperinsulinemia can reduce cell apoptosis and induce cell proliferation through insulin and the insulin-like growth factor-1 (IGF-1) pathway [14]. It has been observed that insulin receptors are overexpressed in most thyroid tumors as an early step in thyroid carcinogenesis [15]. Moreover, in several studies, hyperinsulinemia and insulin resistance were significantly associated with the carcinogenesis and aggressiveness of thyroid cancer [16,17,18].

## 3. Overview of GLP-1 Receptor Agonists

### 3.1. Mechanism of Action

**Figure 1:**
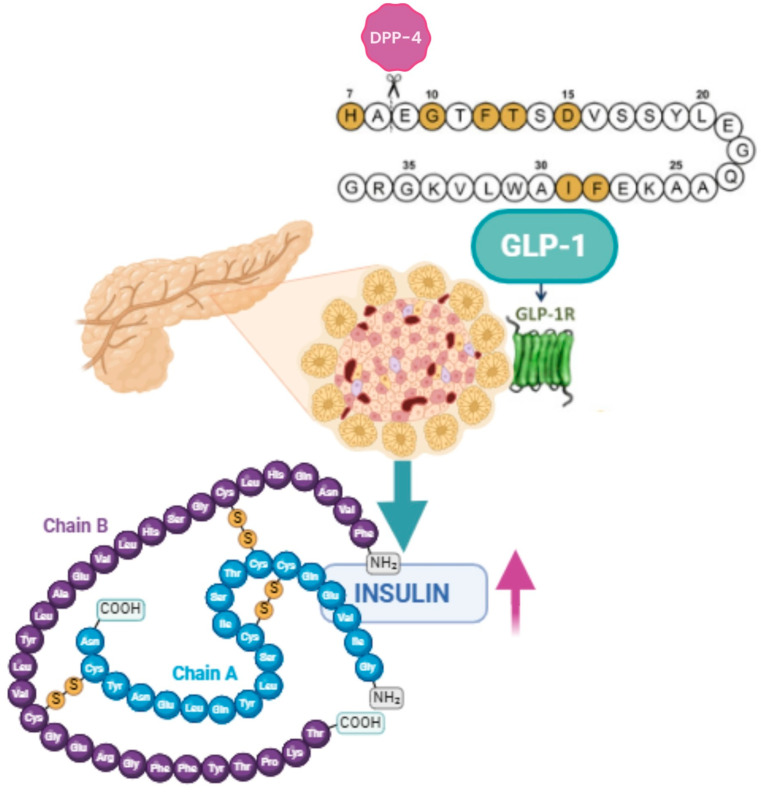
GLP-1 facilitates insulin secretion (BioRender.com). Dipeptidyl peptidase 4 (DPP-4) metabolizes GLP-1 by cleaving the peptide chain between Ala-8 and Glu-9.

Therapies acting on GLP-1R exert their effect on glucose control through multiple mechanisms based on the incretin effect that delineates the occurrence wherein oral glucose evokes a greater insulin secretion compared with intravenous glucose despite inducing similar levels of glycemia in healthy individuals. This phenomenon is orchestrated by the enteroendocrine hormones GLP-1 and Gastric Inhibitory Peptide (GIP), both of which concomitantly facilitate insulin secretion (Figure 1). This effect exhibits a consistent impairment in individuals diagnosed with T2DM [19]. GLP-1 and GIP are “incretin” hormones that are released during a meal, after the ingestion and absorption of glucose, protein, and fat, and provide one of the physiologic connections between eating and insulin release [20,21]. 

**Figure 2:**
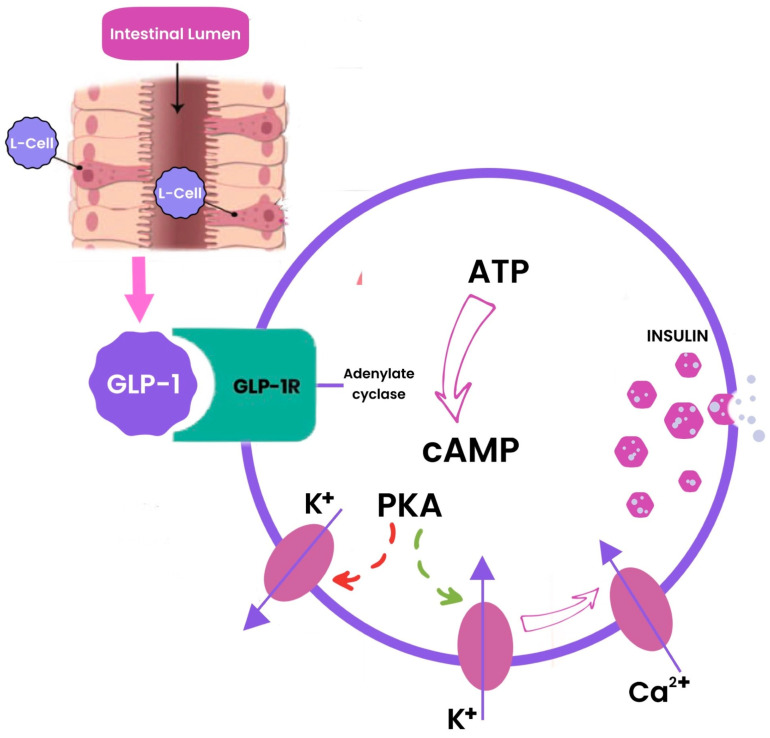
Schematic illustration of the intracellular signaling cascade where GLP-1 stimulates insulin release in beta-cells.

GLP-1, produced in the intestinal epithelial endocrine L-cells by differential processing of proglucagon [22], generates physiological responses by binding with GLP-1R, which is a class B1 (secretin-like family) seven transmembrane spanning, heterotrimeric G-protein coupled receptor [23], located on specific target cells. The stimulation of GLP-1R initiates an intricate intracellular signaling cascade, culminating in the activation of the protein kinase A (PKA) pathway through the generation of cyclic adenosine monophosphate (cAMP) (Figure 2). In addition to the pancreas, GLP-1R is distributed in various tissues, including the lungs, kidneys, central nervous system, stomach, cardiomyocytes, and vascular endothelial cells [20]. 

Other studies have demonstrated the presence of GLP-1R in hepatocytes [24], in thyroid cells, and in thyroid C cells [25]. For example, He et al. [26] conducted a study examining the expression of insulin receptor (IR), IGF-1 receptor (IGF-1R), and GLP-1R in normal thyroid tissue, papillary thyroid carcinoma (PTC) tissues, and PTC cells as well as the potential role of insulin analogs and GLP-1 RAs in cell proliferation and energy metabolism. All three receptors were detected in both PTC tissues and PTC cell lines, as well as in normal thyroid cells. GLP-1R was found to be overexpressed in human PTC tissues/cells, and the expression of IGF-1R and GLP-1R was more pronounced in PTC compared to normal thyroid cells. Researchers investigated cell proliferation, levels of phosphoinositide 3-kinase/AKT serine/threonine kinase (Akt) and mitogen-activated protein kinase/extracellular signal-regulated kinase (Erk) signaling pathway members, and metabolic activity of cell lines exposed to GLP-1 RAs, concluding that GLP-1 RAs may not influence cell proliferation or energy metabolism in PTC cells. Thus, there is currently no need to avoid the use of these antidiabetic agents in patients with PTC. 

Another study by Boess et al. [27] investigated primary cultures of rat and human thyroid cells to assess the expression and function of GLP-1R in C cells. In this study, GLP-1R expression was observed in primary rat C cells but was not detected in primary human C cells. Stimulation with GLP-1 RAs resulted in a modest increase in calcitonin release and expression in primary rat thyroid cultures; however, no functional response to GLP-1 RAs was observed in human thyroid cultures. This lack of functional response of human cultures to GLP-1 RAs suggests that human C cells have very low levels or completely lack functional GLP-1R. Conversely, using primary rat thyroid cells in culture, mRNA expression, and a functional response were observed after stimulation with GLP-1 RAs. These results further support that GLP-1 RAs-induced responses in C cells in rodents may not be relevant to humans. However, it cannot be ruled out that the lack of response to GLP-1 RAs in human primary thyroid cultures is due to the low percentage of C cells and very low calcitonin release, which is not detectable with the methods used in this study. However, despite the studies conducted, the function of the receptors on these cells is not yet fully understood.

### 3.2. Clinical Indications

The first therapeutic GLP-1 RA demonstrating clinically relevant efficacy was exendin-4, a native peptide hormone isolated from the salivary secretion of the Gila monster (Heloderma suspectum). Exendin-4, manifesting comparable activity to GLP-1, exhibited an extended half-life attributable to its heightened resistance against dipeptidyl peptidase 4 (DPP-4), ascribed to the substitution of Ala-8 by Gly-8 at the cleavage site. The Food and Drug Administration (FDA) sanctioned exenatide in 2005 in parenteral formulations as an adjunct to diet and exercise for the amelioration of blood glucose levels in adults with T2DM. 

Numerous studies have investigated the impact of GLP-1 RAs on body weight. A notable meta-analysis conducted by Yao et al. [28] evaluated and compared the efficacy of 16 different GLP-1 RAs in terms of glycemic control, body weight, and lipid profiles in adults with T2DM. Specifically, for the evaluation of changes in body weight, 53 studies involving 21,349 participants were included. The analysis concluded that the combination of cagrilintide and semaglutide was the most effective GLP-1 RAs in reducing body weight. Additionally, tirzepatide, retatrutide, orforglipron, semaglutide, and liraglutide also showed significant weight loss effects compared to placebo.

In another recent systematic review [29], the effects of semaglutide on body weight were evaluated in comparison to other GLP-1 RAs, including liraglutide, exenatide, dulaglutide, and, more recently, the dual GLP-1/GIP receptor agonist tirzepatide. The findings from this review suggest that semaglutide induces a weight reduction of approximately 1–2% every 10 weeks in patients with T2DM and is more effective than other GLP-1 RAs, with the exception of tirzepatide, which is more efficacious. Weight loss is often more significant with higher doses of semaglutide, with 1.0 mg subcutaneously being the most effective regimen without an increase in adverse effects.

Currently, various formulations of GLP-1 RAs are available, with administrations ranging from once daily (such as lixisenatide and liraglutide) to twice daily (exenatide bid) or even once a week (dulaglutide, albiglutide, and semaglutide). A daily oral formulation of semaglutide has recently been approved, demonstrating effective clinical outcomes [30]. 

Since 2016, numerous studies have substantiated the effectiveness of GLP-1 RAs in reducing the mortality associated with critical cardiovascular events, such as acute myocardial infarction and stroke. GLP-1 RAs are particularly suitable for use in combination with metformin (and/or other oral agents) in specific contexts, such as the presence of atherosclerotic cardiovascular disease (ASCVD), elevated glycated hemoglobin levels, primary goals of body weight loss, or avoiding hypoglycemia. In patients with chronic kidney disease, preference is generally given to sodium-glucose co-transporter 2 (SGLT2) inhibitors, although a GLP-1 RA may be considered if SGLT2 inhibitors are contraindicated or if further glucose lowering is needed [31,32,33]. 

Beyond its impact on pancreatic and gastric function, GLP-1 is capable of influencing the brain to induce satiety and reduce food intake. Previous studies have identified the expression of GLP-1R in the hypothalamic nuclei, medulla oblongata, and parietal cortex of humans, consistent with findings in rodents and primates [34].

The prevalence of obesity has significantly increased in recent times, and currently, there are few drugs with proven safety and efficacy for its treatment. This has prompted studies evaluating the effect of liraglutide on body weight and tolerability in individuals with obesity without T2DM. In the study conducted by Astrup et al., participants treated with liraglutide experienced a significant weight loss compared with those treated with a placebo, accompanied by improvements in some obesity-related risk factors [31].

The mechanisms by which GLP-1 RAs induce weight loss are multifaceted and are linked to GLP-1R activation in both central and peripheral enteric neurons, slowing gastric emptying and intestinal transit, as well as suppressing appetite at the hypothalamus level, collectively influencing a mechanism known as the “ileal brake”. A complex gut-brain relationship is evident, as satiety is influenced by GLP-1R activation present in both the central nervous system while the intestine is further modulated by vagal cholinergic signals. It has been suggested that, centrally, the modulation of anorexigenic proopiomelanocortin (POMC) neurons is responsible for the satiety effects, as they possess GLP-1R and are found in the nucleus of the solitary tract (NTS), a site of GLP-1 production in the brain [35].

Currently, liraglutide and semaglutide are the only two pharmacological agents among GLP-1 RAs indicated, in conjunction with a low-calorie diet and regular physical activity, for the management of body weight in adults with a BMI exceeding 30 kg/m^2^ or falling within the range of 27 to <30 kg/m^2^, in the presence of at least one weight-related co-morbidity. These co-morbidities may include dysglycemia, hypertension, dyslipidemia, or obstructive sleep apnea.

### 3.3. Safety Profile

Beyond glycemic control, GLP-1 RAs exhibit a positive influence on various cardiometabolic risk factors, diminishing the likelihood of cardiovascular events and cardiovascular mortality [36].

Nevertheless, a comprehensive understanding of the prolonged safety profile of GLP-1 RAs is yet to be conclusively established. The adverse effects associated with GLP-1-based therapies predominantly manifest in the gastrointestinal tract with symptoms such as nausea, vomiting, and diarrhea. The challenge of gastrointestinal tolerability has the potential to impact patient adherence. Nevertheless, these effects seem to primarily range from mild to moderate severity, with a tendency to diminish over time. The implementation of a gradual titration program has shown promise in alleviating or preventing nausea. Nausea, vomiting, and diarrhea may also manifest in cases of gastroparesis or severe gastroesophageal reflux disease. Existing guidelines emphasize the importance of vigilant monitoring for patients with these conditions when treated with GLP-1 RAs [37]. An assessment of conceivable safety and tolerability concerns linked to GLP-1 RAs has indicated a generally low risk of hypoglycemia, injection-site reactions, pancreatitis, neoplasms, and gallbladder events. 

Furthermore, preclinical investigations have hinted at a potential correlation between the use of GLP-1 RAs and an elevated susceptibility to thyroid C cell tumors in rodent models. Contraindications to the use of GLP-1 RAs regard individuals meeting certain criteria, including those with a personal or familial history of medullary thyroid carcinoma (MTC) or diagnosed with multiple endocrine neoplasia syndrome type 2. Additionally, individuals who have experienced a severe hypersensitivity reaction are advised against its use [38].

## 4. Effect of GLP-1 RAs on the Hypothalamus-Pituitary-Thyroid Axis

The literature suggests that GLP-1R activation may have an impact on thyroid hormone levels and, more generally, on thyroid function parameters, although the exact mechanism and outcomes are still being elucidated. However, several studies reported contrasting findings, with a decrease in thyroid-stimulating hormone (TSH) levels as a result of GLP-1R activation [39,40,41].

Sencar et al. [39] conducted a study on 46 diabetic patients treated with exenatide and found a significant reduction in serum TSH concentration without a significant change in free thyroxine (fT4), free triiodothyronine (fT3), and calcitonin levels before and after treatment. The same result was found in 39 obese diabetic patients treated with exenatide for 6 months in the Köseoğlu et al. study [40], with no significant changes in fT3 and fT4 levels over time. Ye et al. [41] demonstrated in a population of 49 diabetic patients with nonalcoholic fatty liver disease (NAFLD) that treatment with liraglutide reduced TSH levels and improved hepatic thyroid hormone resistance, which is typical of NAFLD patients.

GLP-1 RAs may have a direct central inhibitory effect, as GLP-1Rs are expressed in the paraventricular nucleus (PVN) of the hypothalamus where thyrotropin-releasing hormone (TRH)-producing neurons are located. This suggests that GLP-1 RAs may directly influence the activity of TRH-producing neurons in the hypothalamus [42].

The impact of GLP-1 on the hypothalamus–pituitary–thyroid (HPT) axis involves complex interactions. While the precise mechanism is not fully understood, the literature suggests that GLP-1Rs may modulate thyroid hormone production and secretion through their effects on various metabolic pathways, such as the central regulation of appetite and energy balance. 

Ruska et al. [43] have explored the relationship and interaction of the GLP-1 system and TRH-producing neurons of hypothalamic PVN in male mice. The results reported that GLP-1R activation has multiple effects on TRH-producing neurons, suggesting a complex interplay between GLP-1 pathways and the regulation of TRH-producing neurons; on the one hand, GLP-1R activation directly stimulates TRH-producing neurons in the PVN; on the other hand, GLP-1R activation may also inhibit them, either by enhancing their inhibitory inputs or by directly inhibiting their axon terminals in the median eminence. Despite these effects on TRH neurons, the study found that GLP-1 RAs induced regulation of TRH-producing neurons in vivo that did not appear to determine the weight loss induced by GLP-1 RAs. This implies that while GLP-1R activation may influence thyroid function through its effects on TRH neurons, other effects, such as the slowing of gastric emptying, are more involved in weight loss. Endogenous GLP-1 may modulate the release of TSH from the pituitary gland, thereby influencing the HPT axis. The mechanism underlying this effect may involve direct or indirect actions of GLP-1 on TSH-secreting cells by the anterior pituitary gland. High affinity binding sites for GLP-1 were identified in the rodent thyrotropic cell line alpha-TSH that were linked to increases in cAMP production [44].

Additionally, the reduction in serum TSH levels is unlikely to be due to a direct effect on the thyroid gland itself because GLP-1Rs are primarily expressed in the C cells of the thyroid, which are not responsible for thyroid hormone synthesis [25]. The variation in serum TSH levels could be linked to weight loss. Indeed, obesity is associated with an increase in serum TSH levels [45,46]. 

However, not all studies have found a link between weight reduction and serum TSH levels. Sencar et al. did not find a significant correlation between weight loss and serum TSH levels [39]. 

Conversely, Tee et al. [47] found a significant but small (−0.23 IU/L) decline in mean TSH levels but no decrease in fT4 levels in obese men and women with T2DM who lost 6.5% body weight following treatment with exenatide for 12 months. Their analysis revealed that individuals who did not experience weight loss while receiving exenatide therapy did not show alterations in serum TSH levels. Based on these findings, the study demonstrated that the decrease in body weight is the primary factor influencing the observed changes in serum TSH levels, without any change in fT4 levels. This effect is likely attributed to an enhanced responsiveness of the hypothalamus and/or pituitary gland to TH. The underlying mechanism of how weight loss changes serum TSH levels remains unclear.

A brief mention in this context should be made of the euthyroid sick syndrome, also known as non-thyroidal illness syndrome, commonly characterized by low total T3 and fT3 levels with low or normal levels of T4 and TSH. This condition is often seen in patients with significant weight loss, severe critical illnesses, calorie deprivation, and following major surgeries. [48],therefore, the weight loss induced by GLP-1 RAs could potentially contribute to the development of this syndrome.

In conclusion, the evidence regarding the effects of GLP-1 on thyroid function is limited and controversial; indeed, other studies suggest that the activation of GLP-1R does not influence thyroid hormone levels [40,49].

## 5. Relationship between GLP-1 and Thyroid Tumors

In recent years, several studies have been conducted with the aim of establishing the expression pattern of GLP-1R in various tissues, specifically in the human thyroid. Studies on both rats and humans have demonstrated the expression of GLP-1R in specific tissue compartments of the pancreas, gastrointestinal tract, brain, lungs, heart, kidneys, and thyroid. The impetus for assessing GLP-1R expression at the thyroid level, with a specific focus on thyroid C cells, stems predominantly from the observed elevation in the incidence of MTCs in toxicological screening studies involving rodents treated with GLP-1 RAs [50]. In particular, as reported by Körner et al. [51], the incidence and density of GLP-1R expression in the thyroid of rodents was significantly higher when compared with humans; furthermore, Bjerre Knudsen et al. [52] highlighted notable species-specific variations in the expression and functional activity of GLP-1R in the thyroid.

Vahle et al. conducted a study to assess whether dulaglutide affected the mass of C cells in monkeys, injecting dulaglutide or a control vehicle for 52 weeks. They evaluated baseline and calcium-stimulated serum calcitonin concentrations at 3, 6, 9, and 12 months. At the end of the study, they concluded that administering dulaglutide at 8.15 mg/kg twice a week for 52 weeks did not increase serum calcitonin in monkeys, nor did it influence thyroid weight, histology, C cell proliferation, or absolute/relative volume of C cells. This confirms that non-human primates are less sensitive than rodents to the induction of proliferative changes in thyroid C cells by GLP-1 RAs [53]. 

An interesting study used immunofluorescence to assess the expression of GLP-1R in thyroid tissue samples, including MTC, C cell hyperplasia, PTC, and normal human thyroid cells. The study identified the expression of these receptors in 18% of papillary thyroid carcinomas and in C cells in 33% of control thyroid lobes. Therefore, according to this study, GLP-1R expression is common in MTC and C cell hyperplasia. Additionally, it is not uncommon in normal C cells, and the expression of GLP-1R was also noted in C cell hyperplasia [25].

In 2014, a study was conducted to evaluate the expression of GLP-1RPTC. The immunohistochemical analysis for GLP-1R revealed immunoreactivity in 32.1% of PTC cases. All cases of MTC showed cytoplasmic expression of GLP-1R. Nodular hyperplasia demonstrated immunoreactivity in 28.6% of cases. Conversely, all normal thyroid follicular cells exhibited a lack of immunoreactivity. Furthermore, there was a nearly significant positive association between the extrathyroidal extension of thyroid tumors and GLP-1R expression [54].

### Clinical Studies of GLP-1 RAs and Thyroid Cancer

In the LEADER study, variations in serum calcitonin levels were systematically examined in patients administered liraglutide and placebo. Over 36 months, calcitonin levels did not increase in either males or females with T2DM treated with liraglutide or placebo. Furthermore, instances of C cell hyperplasia or MTC were not observed in patients subjected to liraglutide treatment [55].

The study conducted by Bezin et al. [56] presents a captivating investigation using a nested case-control analysis utilizing the French National Healthcare Insurance System (SNDS) database. The cohort included individuals diagnosed with T2DM who were treated with second-line antidiabetic drugs from 2006 to 2018. Identification of thyroid cancer cases relied on hospital discharge diagnoses and medical procedures carried out between 2014 and 2018. The exposure to GLP-1 RAs was scrutinized within the 6 years preceding a 6-month lag-time period, distinguishing between current use and cumulative duration based on defined daily doses (≤1, 1 to 3, >3 years). Case subjects were meticulously matched with up to 20 control subjects, considering variables such as age, sex, and the length of diabetes, using the risk-set sampling procedure. The risk of thyroid cancer associated with GLP-1 RA use was estimated through conditional logistic regression. The analysis included adjustments for variables such as goiter, hypothyroidism, hyperthyroidism, other antidiabetic drugs, and the social deprivation index. This comprehensive study encompassed 2562 case subjects with thyroid cancers, systematically matched with 45,184 control subjects. Notably, the use of GLP-1 RAs for 1–3 years demonstrated an association with an increased risk of both all thyroid cancer (adjusted hazard ratio [aHR]—1.58, 95% confidence interval [CI] 1.27–1.95) and MTC (aHR 1.78, 95% CI 1.04–3.05), albeit with the limitations of an observational study, with the lack of data about family history of thyroid cancer and exposure to environmental radiation. 

In a recent study conducted by Zhang et al., the safety of GLP-1 RAs, such as liraglutide, in the treatment of T2DM was investigated, with a specific focus on potential thyroid-related risks. The study delved into the molecular mechanisms underlying thyroid diseases induced by GLP-1 analogs, using thyroid cancer cell lines that express GLP-1R. In contrast to previous rodent studies, the results indicated that GLP-1R activation inhibited the proliferation and migration of human thyroid cancer cells. Additionally, the study highlighted the differential response to GLP-1 RAs between rodents and humans. Specifically, the findings suggested that liraglutide might not accelerate cell proliferation in humans and, instead, inhibit Akt and mammalian targets of rapamycin (mTOR) signaling pathways. The study concluded that GLP-1 RA-based treatment, particularly with liraglutide, could be beneficial for managing comorbid diabetes and thyroid cancer, emphasizing the need for further research to address safety concerns raised by rodent experiments and validate the therapeutic potential of liraglutide in this context [57].

A study conducted by Mali et al. analyzed all reports of thyroid cancer associated with GLP-1 analogs in the EudraVigilance database from their initial marketing authorization until 30 January 2020. During the study period, there were 11,243 cases of thyroid cancer and related preferred terms (PTs) among the 6,665,794 reports recorded in the EudraVigilance database. GLP-1 analogues were implicated in 236 cases. Exenatide, liraglutide, and dulaglutide met the criteria for generating a safety signal, indicating that thyroid cancer is reported relatively more frequently in connection with these drugs compared with other medicinal products. The association was most significant for liraglutide, followed by exenatide. According to the authors, this study provides evidence that GLP-1 RAs are associated with thyroid cancer in patients with diabetes [58].

A very recent narrative review by Espinosa De Ycaza et al. [59] analyzed and described the entirety of existing evidence on the association between the use of GLP-1 RAs and thyroid cancer, highlighting that while there is biological plausibility supporting an association between GLP-1 RAs and MTC in rodents, this association is less unequivocal for non-MTC tumors in humans. As per this review, clinical evidence emanating from randomized controlled trials (RCTs) and their attendant meta-analyses underscores that the occurrence of thyroid carcinoma is relatively infrequent, thereby engendering imprecision in effect estimations and consequently a paucity of conclusive and consistent evidence regarding an elevated risk among GLP-1 RAs recipients. Observational studies, which inherently harbor a heightened susceptibility to bias, similarly manifest diminished incidence rates for thyroid carcinoma, with effect estimations evincing incongruities across diverse investigations. Conversely, pharmacovigilance studies consistently show an increase in the reporting of thyroid cancer in patients treated with GLP-1 RAs. Overall, the authors conclude that there is no conclusive evidence of a high risk of thyroid cancer in subjects treated with GLP-1 RAs.

Bea et al. [60] conducted a population-based cohort study utilizing data from compensation claims in the Korean National Health Insurance database from 2014 to 2020, creating two distinct cohorts to compare each incretin-based drug with SGLT2 inhibitors, chosen as active comparators due to their prior non-association with thyroid cancer. The researchers found no association between the use of GLP-1 RAs and thyroid cancer (weighted HR [wHR] 0.98, 95% CI 0.62–1.53) compared to that of SGLT2 inhibitors. Additionally, the researchers highlighted that the use of DPP-4 inhibitors was also not associated with an increased risk of thyroid cancer (wHR 0.95, 95% CI 0.79–1.14) compared to that of SGLT2 inhibitors.

Another recent systematic literature review conducted by Feier et al. [61] analyzed the incidence of thyroid cancer and the detailed spectrum of adverse events associated with semaglutide. Among the 10 studies analyzed, the incidence of thyroid cancer was notably low, with a few isolated cases of PTC and MTC reported, each constituting less than 1% within the respective study groups, suggesting the absence of a significant risk of thyroid cancer associated with semaglutide use considering the large sample sizes.

Silverii et al. [62], with a meta-analysis of RCTs examining 64 studies, wanted to investigate the association between treatment with GLP-1 RAs and the onset of thyroid cancer. Treatment with GLP-1 RAs was found to be significantly associated with an increased overall risk of thyroid carcinoma (Mantel–Haenszel odds ratio [MH-OR] 1.52 95% CI 1.01–2.29; *p* = 0.04, I^2^ = 0%). However, the authors did not find any significant association for PTC (MH-OR 1.54, 95% CI 0.77–3.06; *p* = 0.22) or MTC (MH-OR 1.44, 95% CI 0.23–9.16; *p* = 0.55). In conclusion, this meta-analysis showed that treatment with GLP-1 RAs may be associated with a moderate increase in the relative risk of thyroid cancer with a small increase in absolute risk.

According to these observations, it emerges that the assessment of the association between the use of GLP1-RAs and the onset of thyroid tumors is complex and subject to varied interpretations. While some studies confirm the expression of GLP-1R in the thyroid and suggest diversified effects across species, the currently available evidence does not allow for definitive conclusions regarding a significant risk of thyroid tumors associated with the use of GLP1 RAs (Table 1). In light of these results, it would be appropriate to carefully evaluate the introduction of GLP-1 RAs in therapy in people with diabetes who have a family history of thyroid tumors and possibly subject such patients to close thyroid monitoring. Results vary among studies, highlighting the need for further research to fully clarify the extent of this association and provide clearer indications of clinical implications. Species differences, variations in cellular response, and methodological limitations in different studies contribute to the complexity of the picture.

## 6. Effect of GLP1-RAs on Thyroid Volumes

Over time, an extensive series of studies has examined the intricate relationship between GLP-1 analogs and thyroid tumors, constituting a substantial body of research, albeit with somewhat controversial results. In contrast, a relatively limited body of academic literature has been devoted to exploring the subtle repercussions of these pharmacological agents on both thyroid volume and function. Some evidence has shown the presence of GLP-1R not only in C cells and MTC cells but also in their expression in normal thyroid tissue and PTC. This complexity of findings has generated various hypotheses, suggesting that such pharmacotherapeutic agents might exert appreciable effects on thyroid volume and, consequently, on its functional dynamics. Köseoğlu et al. conducted a prospective study with the aim of examining the effects of a 6-month treatment with exenatide on the structural and functional characteristics of the thyroid in obese patients with T2DM. All participants in this study had a body mass index (BMI) exceeding 35 kg/m^2^ and had not previously used exenatide prior to study enrollment. All patients underwent laboratory analyses before treatment initiation and after 6 months of study participation, coupled with thyroid ultrasound for gland volume calculation, computed as “volume (mL) = length (cm) × width (cm) × depth (cm) × 0.524”. The study results revealed that 6 months of exenatide treatment reduced thyroid volume and TSH levels, whereas nodule dimensions and echogenicity, along with levels of fT3, fT4, calcitonin, and CEA, remained unchanged over time. TSH is a primary thyroid proliferation factor, and the reduction in TSH may contribute to a decrease in thyroid volume. However, regression analysis did not demonstrate any correlation between the reduction in TSH and a decrease in thyroid volume [40]. Further, analyses could also be conducted on the possible relationship between GLP-1 RAs use and serum thyroglobulin (Tg) levels, as some studies have shown that Tg correlates with abnormal thyroid function and thyroid volume [63].

An additional observational study conducted in a single center involving 46 diabetic patients without thyroid disease aimed to elucidate the relationship between GLP-1 RAs and thyroid volume in individuals with T2DM but without any pre-existing thyroid pathology. In stark contrast to the previously mentioned study, the results of this investigation demonstrated that a 6-month treatment with exenatide did not elicit statistically significant changes in thyroid volume. However, it did lead to a reduction in TSH levels, in agreement with the findings of the study conducted by Köseoğlu et al.; however, noteworthy limitations of this study include the relatively brief duration of the exenatide treatment and the absence of a control group. The outcomes of this exploration into the interaction between GLP-1 RAs and thyroid dynamics in diabetic patients without pre-existing thyroid pathologies reveal contrasting results between the studies. Particularly in this case, similar results have been obtained regarding the reduction of TSH levels, while opposite results have been obtained regarding thyroid volume, even though it should be taken into consideration that the baseline thyroid volume was greater in the study conducted by Köseoğlu et al. (Table 2). Therefore, it is essential to emphasize the importance of further comprehensive investigations into the impact of GLP-1 RAs on thyroid function and structure in individuals with diabetes [39].

## 7. Conclusions

This review has provided a thorough analysis of the relationship between the use of GLP-1 RAs and thyroid function in patients with T2DM. Through the synthesis of available evidence and the investigation of knowledge gaps, we aimed to elucidate the complex interaction between these molecules and thyroid structure and function.

In conclusion, despite conflicting results in the literature, the importance of carefully evaluating thyroid function in diabetic patients, particularly those treated with GLP-1 RAs, is evident. The profound interconnection between thyroid dysfunction and metabolic conditions underscores the significance of an integrated management approach for these pathologies. This review highlights the necessity of implementing routine thyroid function screenings in diabetic patients undergoing treatment with GLP-1 RAs, aiming to optimize clinical and healthcare management; for example, it may be beneficial to include thyroid function tests, particularly TSH measurement and an annual thyroid ultrasound in people with diabetes’ routine tests. Furthermore, in patients with a family history of MTC, it would be useful to measure calcitonin annually. Nowadays, guidelines still do not give precise indications on how to behave in these situations and in patients at greater risk of thyroid disorders, as the evidence is still insufficient to draw conclusions on the effects of GLP-1 RAs on the thyroid. Additionally, such an approach could provide a more comprehensive understanding of the thyroid health of diabetic patients and enable early intervention in cases of thyroid function abnormalities. The importance of further studies to better understand the role of GLP-1 RAs on thyroid function and to delineate the underlying mechanisms of this complex interaction is evident. 

## Figures and Tables

**Table 1:** Summary of clinical evidence about GLP-1 RAs and the risk of developing thyroid cancer. RCT: randomized clinical trial; ETR: estimated treatment ratio; CI: confidence interval; MTC: medullary thyroid cell carcinoma; aHR: adjusted hazard ratio; PRR: proportional reporting ratio; GLP-1 RAs: Glucagon-like Peptide-1 receptor agonists; aHR: adjusted hazard ratio; PTC: papillary thyroid carcinoma; SGLT2i: Sodium-glucose cotransporter 2 inhibitors; wHR: weighted hazard ratio. Green: no increase in the risk of thyroid cancer; red: increased risk of thyroid cancer.

Study	Characteristics of the Study	Findings
LEADER 2018 [55]	RCT liraglutide (*n* = 4364) vs. placebo (*n* = 4321) Follow-up: 3.5–5 years	No increase in calcitonin concentrations in the liraglutide group ○ male: ETR 1.03 95% CI 1.0–1.06, *p* = 0.068 ○ female: ETR 1.00 95% CI 0.97–1.02, *p* = 0.67 No cases of MTC in liraglutide group
Mali et al. 2020 [58]	Case/non-case analysis on report EudraVigilance 11,243 cases of thyroid cancer (236 involved GLP-1 RAs) until 30 January 2020	Exenatide, liraglutide, and dulaglutide met the criteria to generate safety signals for thyroid cancer ○ Liraglutide: PRR 27.5 95% CI 22.7–33.3 ○ Exenatide: PRR 22.5 95% CI 17.9–28.3 ○ Dulaglutide: PRR 13.1 95% CI 9.4–18.3
Bezin et al. 2023 [56]	Nested case–control analysis subjects with thyroid cancer (*n* = 2562) vs. controls (*n* = 45,184)	Increased risk of all thyroid cancer and MTC in subjects receiving GLP-1 RAs for 1–3 years ○ All thyroid cancer: aHR 1.58 95% CI 1.27–1.95 ○ MTC: aHR per MTC 1.78, 95%CI 1.04–3.05
Silverii et al. 2023 [62]	Meta-analysis of 64 RCT GLP-1 RAs (*n* = 46,228) vs. any comparator (*n* = 38,399)	Increased risk of all thyroid cancer in GLP-1 RA users ○ MH-OR 1.52 95%CI 1.01–2.29, *p* = 0.04, I^2^ = 0% No significant association with PTC or MTC
Bea et al. 2023 [60]	Population-based cohort study New users of GLP-1 RAs (*n* = 21,722) vs. new users of SGLT2i (*n* = 326,993)	GLP-1 RAs are not associated with an increased risk of thyroid cancer ○ wHR 0.98 95%CI 0.62–1.53
Feier et al. 2024 [61]	Systematic review of 10 RCT (use of semaglutide) *n* = 14,550 with 7830 receiving semaglutide	Incidence of thyroid cancer less than 1% in semaglutide users à no significant risk

**Table 2:** The relationship between GLP-1 RAs, thyroid volume, and TSH in individuals with T2DM without any pre-existing thyroid pathology. Results obtained from the study by Sencar [39] and Köseoğlu [40].

	TSH before Exenatide (mU/L)	TSH 6 Months after Exenatide (mU/L)	*p*	Thyroid Volume before Exenatide (cm^3^)	Thyroid Volume 6 Months after Exenatide (cm^3^)	*p*
Sencar 2019 [39]	2.3	1.8	0.007	11.6 ± 9.0	12.1 ± 8.8	0.19
Köseoğlu 2020 [40]	2.36 ± 1.17	1.95 ± 0.91	0.007	17.39 ± 15.72	15.72 ± 13.17	0.043

Green indicates concordant results, while red indicates contrasting results. TSH: thyroid-stimulating hormone.

## Data Availability

Not applicable.
